# Cognition and the Placebo Effect – Dissociating Subjective Perception and Actual Performance

**DOI:** 10.1371/journal.pone.0130492

**Published:** 2015-07-06

**Authors:** Katharina A. Schwarz, Christian Büchel

**Affiliations:** Department of Systems Neuroscience, University Medical Center Hamburg-Eppendorf, Hamburg, Germany; University G. d'Annunzio, ITALY

## Abstract

The influence of positive or negative expectations on clinical outcomes such as pain relief or motor performance in patients and healthy participants has been extensively investigated for years. Such research promises potential benefit for patient treatment by deliberately using expectations as means to stimulate endogenous regulation processes. Especially regarding recent interest and controversies revolving around cognitive enhancement, the question remains whether mere expectancies might also yield enhancing or impairing effects in the cognitive domain, i.e., can we improve or impair cognitive performance simply by creating a strong expectancy in participants about their performance? Moreover, previous literature suggests that especially subjective perception is highly susceptible to expectancy effects, whereas objective measures can be affected in certain domains, but not in others. Does such a dissociation of objective measures and subjective perception also apply to cognitive placebo and nocebo effects? In this study, we sought to investigate whether placebo and nocebo effects can be evoked in cognitive tasks, and whether these effects influence objective and subjective measures alike. To this end, we instructed participants about alleged effects of different tone frequencies (high, intermediate, low) on brain activity and cognitive functions. We paired each tone with specific success rates in a Flanker task paradigm as a preliminary conditioning procedure, adapted from research on placebo hypoalgesia. In a subsequent test phase, we measured reaction times and success rates in different expectancy conditions (placebo, nocebo, and control) and then asked participants how the different tone frequencies affected their performance. Interestingly, we found no effects of expectation on objective measures, but a strong effect on subjective perception, i.e., although actual performance was not affected by expectancy, participants strongly believed that the placebo tone frequency improved their performance.

## Introduction

Expectancy effects have been extensively investigated in clinical research, especially with regard to placebo hypoalgesia [1–2], and with regard to placebo effects in clinical conditions such as Parkinson’s disease [3] or depression [4]. Originally, these expectation effects on overt behavior, subjective well-being, and physiological measures, have been regarded as a cumbersome confound with the potential to bias clinical research [1, 5]. This view, however, has changed in recent years, and more and more clinicians are called to realize the potential benefit of expectation effects when they are deliberately used to the patient’s advantage (e.g., in combination with an established therapy) [5]. Research on placebo effects can thus be of genuine clinical interest, as is research on the nocebo effect, i.e., negative effects of expectations on physical and subjective well-being [5–7].

Therapies, however, are not only used as treatment for diseases. Instead, the increasing use of drugs supposed to enhance cognitive performance–“cognitive enhancers”–by healthy individuals has stimulated controversial debates [8–12]. No matter their ethical conundrums, it seems as if the use of cognitive enhancers in critical situations is already reality on many university campuses [11–12]. However, the mechanisms and possible side effects in healthy individuals are not very well understood [8, 10, 12]. This raises the question if placebo effects could not be part of the picture—and maybe even part of the solution. Is it possible to elicit performance improvements simply by evoking the *expectancy* of situational performance improvement? And is it possible to induce the opposite, a cognitive impairment, simply by suggesting that such performance impairment should take place?

Several factors speak in favor of this possibility: For one, similar expectancy effects have been described in the extensive literature on placebo effects in various domains [5]. For another, cognitive performance is susceptible to a range of social expectancy effects, such as stereotype threat or self-efficacy effects [13–15], suggesting that expectancy effects are a ubiquitous phenomenon. In relation to cognitive enhancement, expectancy effects affected objective measures when participants expected to receive methylphenidate, a well-known cognitive enhancer, but received placebos instead. These participants showed altered blood-oxygen-level dependent (BOLD) brain responses, e.g., in the nucleus accumbens, a brain area associated with the processing of reward, and reported higher subjective restlessness and “drug liking” compared to a condition in which they expected and received placebo treatment [16].

However, the most consistent placebo effects have been found in regard to subjective states, not objective measures [17]. For example, the expectation to receive methylphenidate affected arousal ratings in participants (“feeling high” and “feeling stimulated”), but it did not improve cognitive performance—actually, it seemed to *impair* cognitive performance in some instances [18]. Please note, though, that no subjective measures regarding perceived cognitive performance were included in this study and participants were specifically chosen based on high-risk factors for stimulant misuse, i.e., it is unknown whether the findings of this study are specific to individuals who endorse such risk factors or transferable to the general population [18]. As another example, placebo treatment in asthma patients led to no change in actual objective physiological parameters compared with a no-intervention control condition; in contrast, a large objective drug effect was found when using a real bronchodilator as treatment [19]. Interestingly, the patients’ subjective perception of symptom improvement was similar for the bronchodilator and the placebo treatment, and both conditions significantly differed from the no-intervention control. These findings indicate that placebo effects, while certainly affecting objective measures in some domains [2, 17], might have very little effect on objective measures in others. Furthermore, the *subjective experience* seems to be largely independent from the objective scores and especially susceptible to expectancy effects.

Whether or not a given domain is susceptible to expectancy effects can only be answered by empirical research using both, subjective and objective measures. In this study, we therefore addressed cognitive performance under conditions of positive or negative expectancies (placebo or nocebo conditions). We further investigated whether potential effects would occur for objective and subjective measures alike or whether they would be mainly restricted to the participants’ subjective perception. To this end, healthy participants completed a Flanker interference paradigm in a placebo, nocebo, and control condition. As expectancy manipulation, we instructed the participants that special tones (i.e., different sound frequencies) were known to differentially affect brain activity and cognitive performance, a phenomenon allegedly called the “frequency stimulation effect”. Before the actual test phase, we induced instruction-congruent experiences by including a conditioning phase adapted from experimental paradigms used in placebo hypoalgesia [20]. This procedure is known to maximize possible expectancy effects, as the literature on placebo hypoalgesia indicates that placebo effects are best elicited when prior experience supports the placebo suggestion [21–22].

## Materials and Methods

### Participants

We recruited 37 individuals (22 female; mean age 25.19 years ± 0.93 SE_M;_ mean age,female participants, 23.86 years ± 0.78 SE_M_; mean age, male participants, 27.13 years ± 1.93 SE_M_) for participation in this study. A power analysis suggested a study sample of at least 34 participants to obtain a power of 80% for an expected effect size of d = 0.50 [17], given statistical analyses by means of two-tailed tests. All participants received payment as compensation. Exclusion criteria involved neurological or neuropsychiatric diseases, current medication, or substance abuse. The study was approved by the Ethics Committee of the Medical Council of Hamburg and all participants gave written consent in accordance with the Declaration of Helsinki.

### Expectancy Manipulation

Participants were informed at the beginning of the experiment that they would take part in a study investigating the effects of “frequency stimulation” on cognitive processes. Frequency stimulation was explained as a method to increase or decrease activity in specific brain areas by hearing sounds of specific tone frequencies. Participants were told that, e.g., higher frequencies would stimulate brain activity and thus improve task performance and lower frequencies would inhibit brain activity and thus impair task performance. A third intermediate frequency would be included to serve as a control stimulus that has no effect on brain activity. The instruction was randomized as to which frequencies (high, intermediate, low) were allegedly designed to increase/decrease brain activity and improve/impair performance or which frequency would have no effect and serve as a control stimulus. All participants were exposed to all sounds to allow for a within-subject comparison between the placebo (“improved performance”), nocebo (“impaired performance”), and control condition.

### Testing Procedure

**Fig 1:**
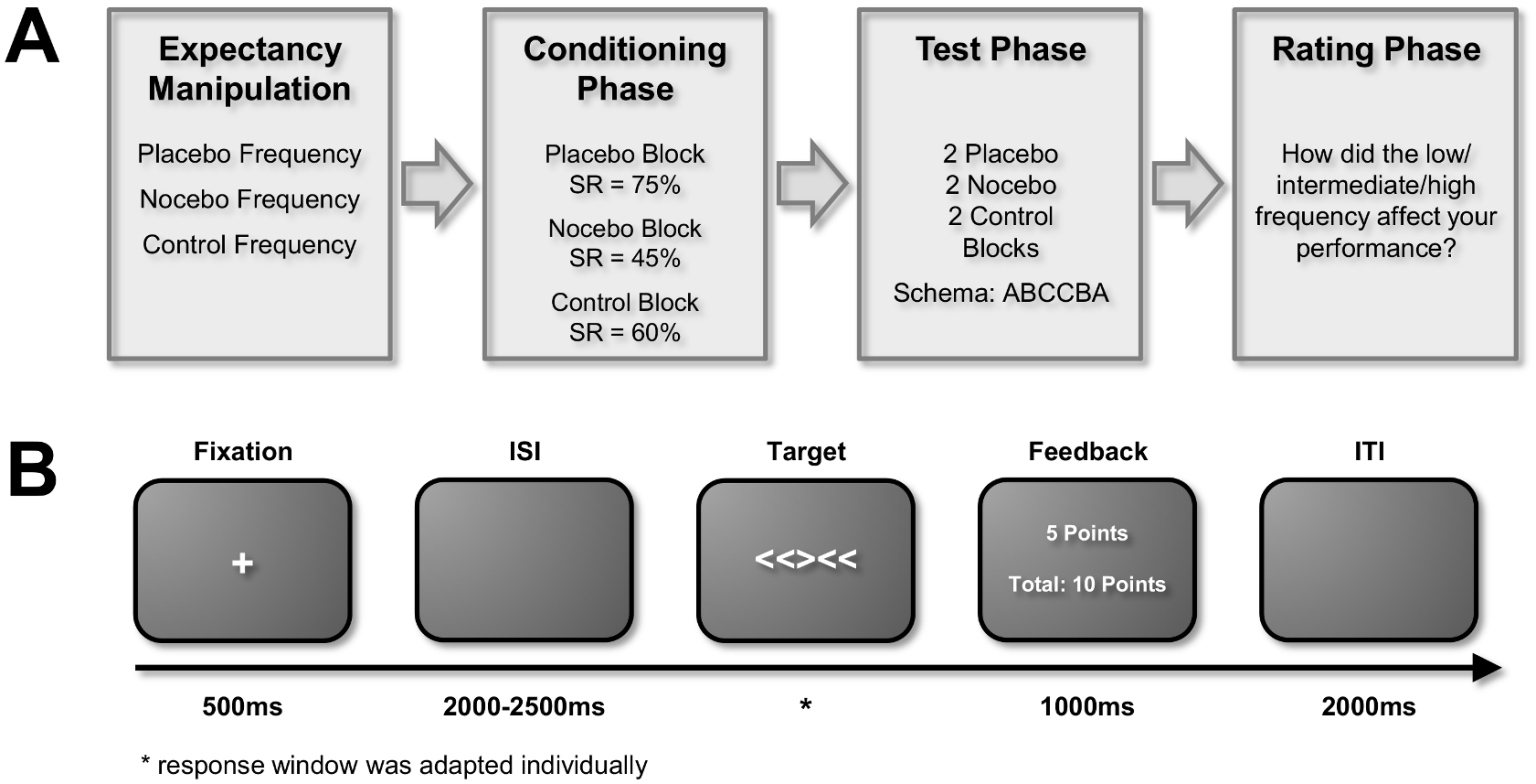
**A. Study design.** The study started with an expectancy manipulation: Participants were first informed about the effects of “frequency stimulation” and heard three different tone frequencies allegedly designed to either improve, impair, or not affect cognitive functioning (placebo, nocebo, and control frequencies). They then underwent a conditioning phase with fixed success rates to strengthen their expectations, followed by the actual test phase without any additional manipulations pertaining to success rates. In the subsequent rating phase, participants evaluated how the frequencies affected their performance. **B. Trial procedure of the Flanker task**. This trial procedure was used during conditioning and to assess cognitive performance during test. Participants first saw a fixation cross on the screen followed by a variable inter-stimulus interval (ISI). Then the actual target appeared; participants were asked to respond with a left or right arrow key press when the middle arrowhead pointed to the left or right, respectively. The response window for this task was adapted individually. If participants responded correctly and in time, they gained 5 points per trial, if not they didn’t gain any points; this information was presented to them together with the total number of points they had gained during the respective block.

After informed consent and the expectancy manipulation, participants first were asked to individually adjust the volume of the different sounds to assure that all sounds were easily audible, but not uncomfortably loud, and that all sounds were perceived as equal in volume. This procedure was intended to account for variability in hearing ability across individuals and across different frequencies within an individual. The participants then underwent a conditioning procedure similar to common paradigms in research on placebo analgesia (Fig 1A) [20]. Such conditioning procedures increase placebo effects by generating personal experience and expectations in line with the expectancy manipulation [21–22]. To measure the participants cognitive performance, they were asked to complete a Flanker task (Fig 1B) in each expectancy condition (placebo vs. nocebo vs. control) while hearing the respective sound frequencies allegedly designed as cognitive enhancers, disrupters, or controls. The order of the condition blocks were randomized across participants. As a conditioning procedure, success rates were fixed at 75%, 45%, and 60% in the placebo, nocebo, and control condition by means of an adaptive staircase algorithm that allowed more or less time to respond to the presented stimuli. At the end of each block, participants received feedback about their performance, i.e., their success rates. Blocks were separated by short breaks.

After the conditioning procedure, participants took part in the actual test phase in which we did not manipulate success rates. The test phase consisted of a single, longer block during which the different expectancy conditions and tone frequencies were presented block-wise according to an ABCCBA schema, i.e., if the participants started with the placebo condition (in this example A), they would also end with the placebo condition, whereas the other expectancy conditions (e.g., nocebo as B and control as C) were placed in between. This procedure was applied to assure that changes in motivation or fatigue would not lead to confounding time effects. Which condition served as condition A, B, or C in this schema was randomized across participants. After the test phase, participants were asked to rate how different tone frequencies affected their performance according to their own opinion. To this end, participants were asked to indicate on a 9-point scale how they thought the different tone frequencies affected their performance (1 –“performance was definitely impaired” to 9 –“performance was definitely improved”). They answered this question once for every frequency used in the experiment.

The whole experimental procedure lasted about 2.5 hours per participant. To assure a high motivation throughout the experiment, we increased the amount of money participants received proportionally to their performance across all experimental tests (including the conditioning and the test phase), and informed the participants about this procedure at the very beginning.

#### Conditioning Phase

All participants first completed a short introductory block of 10 trials to become acquainted with the task. Control of the experimental timing and the stimulus presentation throughout the experiment was achieved using Presentation 16.4, NeuroBehavioral Systems (Albany, CA, USA). Each trial started with a fixation cross presented on a computer screen for 500ms. After a variable inter-stimulus interval (ISI) of 2000 to 2500 ms (sampled from a discrete uniform distribution with steps of 50 ms each), five arrowheads (target stimulus) were presented on the screen pointing either to the left or the right. Participants were instructed to respond with the right arrow key on the computer keyboard when the central arrowhead pointed to the right and to respond with the left arrow key when the central arrowhead pointed to the left, irrespective of the other arrowheads presented. The four arrowheads surrounding the center arrowhead all either pointed in the same direction as the center arrowhead (compatible condition) or in the opposite direction (incompatible condition). The target stimulus was presented until participants responded with a button press but for a maximum duration of 1000 ms. If they answered correctly and in time, a feedback screen told them that they had gained 5 points for the trial; if they did not answer correctly or if they responded too early, i.e., during the ISI, or too late, the feedback screen informed them that they had received 0 points for the trial. The next trial started after an inter-trial interval (ITI) of 2000 ms.

During the conditioning procedure, the participants heard the respective tone frequency during the entire expectancy condition block. Each expectancy condition block started with an additional short introductory block of 20 trials. We used these trials to assess for each participant individually which response window he or she needed to complete 75%, 45% or 60% of the trials successfully in the placebo, nocebo, or control condition, respectively. This information was then fed as the starting point into the staircase algorithm for the actual conditioning phase, i.e., the response window the participants needed during the first 20 trials to complete 60% of the trials successfully was the response window the participants had in the first conditioning trial to respond to the Flanker task target in the control condition. The overall success rate in the actual conditioning block was then calculated after each trial; if the success rate was greater than 75%, 45% or 60% in the respective expectancy conditions, the response window available for the participants to respond to the target was shortened by 10 ms, if the success rate was lower than 75%, 45% or 60% in the respective expectancy conditions, the response window was extended by 10ms. This led to a fixed success rate of 75%, 45% or 60%, respectively, after all 80 trials of the expectancy condition block were completed. The success rate and the absolute number of points were then presented as feedback to the participants. This procedure was conducted for each expectancy condition (placebo, nocebo, and control), with short breaks after each run.

#### Test Phase

The test phase started with an introductory block of 40 trials. The first 20 trials of this block were intended as an opportunity for the participants to get acquainted with the task again, the last 20 trials were used to assess the response window that the participants needed to respond successfully to 60% of the trials. This response window then served as the maximum response window for the remainder of the test phase. This adaptation procedure was again adapted from common paradigms in placebo analgesia (e.g., [20]) After the introductory block, the actual test phase started either with the placebo, nocebo, or control condition and proceeded according to the ABCCBA schema mentioned above. Participants completed six test blocks (two of each condition) à 35 trials each; the blocks were separated by short breaks. The respective tone frequencies were only heard during the test blocks, not during the introductory block before. After all six blocks were completed, the participants again received feedback about the success rate and the absolute number of points they had gained during the test phase. At the end of the experiment, participants were debriefed about the actual study purpose and were asked to indicate on a 7-point scale whether they had believed the cover story. One male participant had to be excluded, because he did not believe our expectancy manipulation.

### Behavioral data analyses

Behavioral data were analyzed using SPSS 20 (IBM, Armonk, NY, USA). For the conditioning phase, we calculated the mean reaction time (RT) of all successfully completed trials for each participant separately for each condition. For the test phase, we calculated success rates for each participant and the mean RT of all successfully completed trials for each participant, separately for each condition. The introductory blocks were not included in the calculations. We then performed an analysis of variance (ANOVA) with the within-subjects factors expectancy (placebo vs. nocebo vs. control) and compatibility (compatible vs. incompatible) for the reaction time and success rate data and performed paired *t*-tests as follow-up analyses. The subjective rating data were also analyzed with an ANOVA (within-subjects factor expectancy) followed by paired *t*-tests.

## Results

In the conditioning phase, we adapted the response window to fix the success rate (SR) to 75%, 60%, and 45% for the placebo, nocebo, and control condition, respectively. Our data indicate that this manipulation was successful (mean SR_placebo_ = 74.44%, mean SR_nocebo_ = 45.35%, mean SR_control_ = 60.31%), *F*(2,70) = 503.18, *p* < .001, η_p_^2^ = 0.93. All follow-up paired *t*-tests showed significant differences between the participants’ SRs dependent on the expectancy condition (*p*s < .001). Even though RT was not specifically manipulated during the conditioning phase, we still found a strong main effect of expectancy, *F*(2,70) = 14.61, *p* < .001, η_p_^2^ = 0.30. This RT effect counteracted the conditioning, with the participants being the fastest in the nocebo condition (374 ms), the slowest in the placebo condition (400 ms), and intermediate in the control condition (388 ms). Again, all follow-up paired *t*-tests confirmed the RT differences between the conditions to be significant (*p*s < .009) and this analysis was further supported by a significant linear contrast placebo > control > nocebo, *F*(1,35) = 28.52, *p* < .001, η_p_^2^ = 0.45. As expected, participants further responded much faster for compatible Flanker stimuli (360 ms) than for incompatible stimuli (433 ms), *F*(1,35) = 422.78, *p* < .001, η_p_^2^ = 0.92 [23], whereas the interaction of expectancy and compatibility was not significant, *F*(2,70) = 2.43, *p* = .105, ɛ = .855 (Greenhouse-Geisser corrected for violations of sphericity).

**Fig 2:**
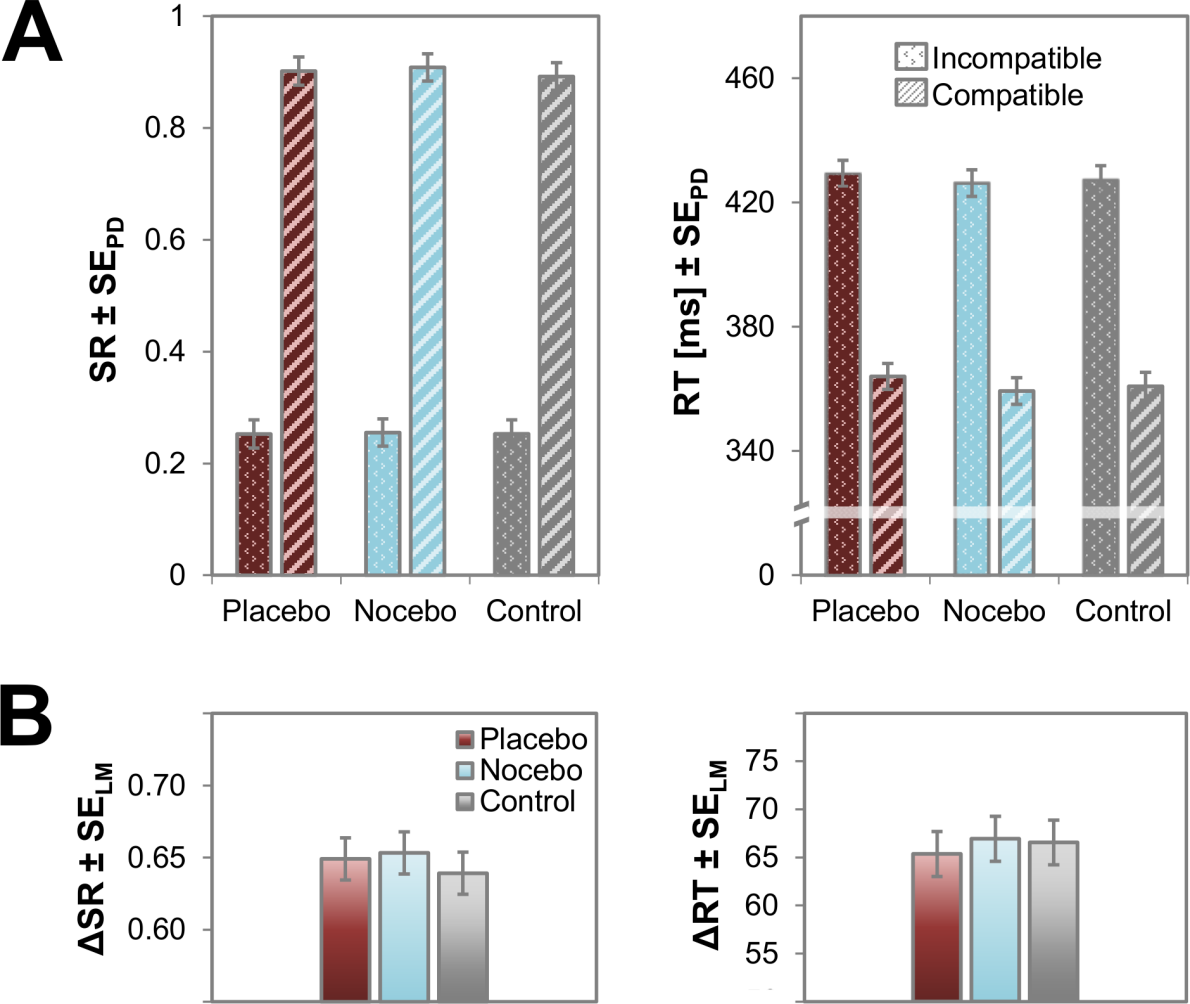
**A.** Success rates (SRs) and reaction times (RTs) as a function of expectancy and Flanker compatibility. Error bars indicate standard errors of paired differences [39], computed separately for each expectancy condition. **B.** Compatibility effects for each expectancy condition, computed as ΔSR = SR_compatible_-SR_incompatible_ and ΔRT = RT_incompatible_-RT_compatible_. Error bars indicate the Loftus-Masson within-subjects standard error for repeated measures ANOVA [40]. Flanker compatibility effects emerged for SRs and RTs, but these effects did not significantly differ between the three expectancy conditions and no main effect of expectancy was significant.

To pinpoint the actual effects of the expectancy manipulation and the corresponding conditioning on objective measures, we analyzed SRs and RTs of the test phase (Fig 2). Our data clearly show that the effects established in the conditioning phase did not carry over to the test phase. More precisely, robust Flanker compatibility effects emerged for SRs, *F*(1,35) = 869.85, *p* < .001, η_p_^2^ = 0.96, and RTs, *F*(1,35) = 293.82, *p* < .001, η_p_^2^ = 0.89, but the effects were virtually identical in size across the three expectancy conditions; SRs: *F*(2,70) = 0.27, *p* = .766, η_p_^2^ = 0.01; RTs: *F*(2,70) = 0.12, *p* = .883, η_p_^2^<0.01. Also, neither main effect of expectancy was significant; SRs: *F*(2,70) = 0.41, *p* = .664, η_p_^2^ = 0.01; RTs: *F*(2,70) = 1.99, *p* = .145, η_p_^2^ = 0.05. To follow up on these analyses, we computed Bayes Factors for the most informative comparison—the difference in compatibility effects between the placebo and the nocebo condition. Bayes statistics allow the computation of the probability of a hypothesis conditionally on observed data. Bayes factors represent the *posterior odds*, the quotient of the probability of the null hypothesis given the observed data and the probability of the alternative hypothesis given the observed data. Among other things, they are thus used to identify whether or not a non-significant effect is due to a statistical power problem or due to an actual absence of a real effect—a distinction which cannot be made with traditional significance testing. Generally, odds greater than 3 are considered substantial evidence for one hypothesis over another. The employed analyses indeed yielded substantial evidence in favor of the null hypothesis of no effect, BF_SR_ = 5.42, BF_RT_ = 4.93, indicating that the above findings indeed reflect the absence of a real effect rather than insufficient power [24].

**Fig 3:**
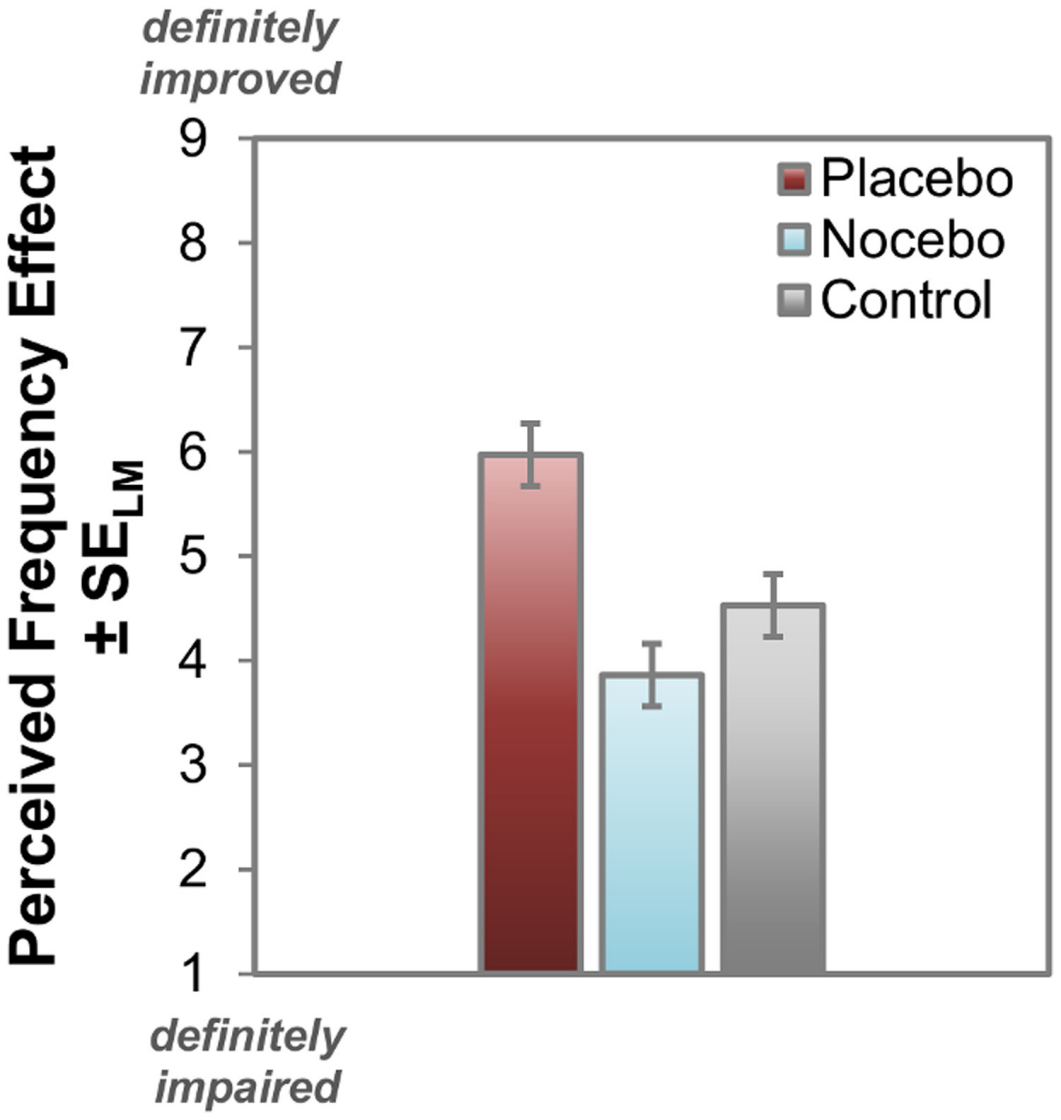
Subjective perception of the frequency effect. Although no frequency effect emerged in objective measures, participants perceived the placebo frequency as having a positive effect on their performance, compared with the control and the nocebo frequency. Error bars indicate the Loftus-Masson within-subjects standard error for repeated measures ANOVA [40]. Participants perceived an effect of frequency on performance. They felt a positive effect of the placebo frequency on their performance compared to both, the control frequency and the nocebo frequency. The difference between the nocebo and the control frequency did not reach significance.

Although no effect of the expectancy manipulation emerged for SRs and RTs, the participants still perceived an effect of frequency on performance as indicated by the subjective rating data (Fig 3), *F*(2,70) = 13.13, *p* < .001, η_p_^2^ = 0.27. Indeed, follow-up paired *t*-tests revealed that participants felt a positive effect of the placebo frequency on their performance compared to both, the control frequency, *t*(35) = 3.36, *p* = .002, d = 0.56, and the nocebo frequency, *t*(35) = 5.93, *p* < .001, d = 0.99. Although the nocebo frequency was descriptively judged to have a worse effect on performance than the control frequency, this difference did not reach significance, *t*(35) = 1.42, *p* = .165. A significant linear contrast (placebo > control > nocebo) further supported the notion that the placebo frequency was perceived as having the most positive effect, followed by the control frequency and the nocebo frequency, *F*(1,35) = 35.20, *p* < .001, η_p_^2^ = 0.50. As a control analysis, we also checked if there was a difference in the rating data for the actual tone frequencies (high, intermediate or low), irrespective of their role in the experiment. Participants did not perceive any particular tone frequency as having a more positive or negative effect on their performance as any other (*F*<1). This finding indicates that the effect in subjective perception depended on the expectancy manipulation, not on the actual frequency of the stimuli.

## Discussion

In this study, we investigated whether placebo and nocebo effects in cognitive tasks can be elicited by evoking positive and negative expectations about own task performance. Expectations were manipulated by instructing participants about alleged effects of different sounds (“frequency stimulation”) on cognitive performance. To maximize possible effects, we implemented a conditioning paradigm adapted from placebo hypoalgesia research, in which participants experienced either high, medium, or low success rates (SRs) in a choice reaction time (RT) task. The effects of the corresponding positive, neutral, or negative expectations were then assessed in the actual test phase in which participants were confronted with tones that had been paired with different SRs. Interestingly, we found no expectancy effects in objective measures of cognitive performance (SR and RT), but a strong effect on subjective perception. Participants were not more or less successful in the respective conditions in terms of actual performance, but they still felt that the experimental manipulation, i.e., the frequency stimulation, affected their performance in line with the previous verbal suggestion and experience during conditioning. To our knowledge, the present study is the first study on cognitive performance to include both, placebo and nocebo instructions into one experimental design in order to investigate expectancy effects on cognitive measures, and to also include a direct subjective measure of perceived cognitive performance. Moreover, the paradigm used was directly adapted from common placebo analgesia paradigms [20], allowing straightforward and informative comparison of our results to studies on placebo analgesia.

Previous literature on expectancy effects in, e.g., asthma patients, but also in the cognitive domain documents similar patterns [18–19]. Since expectancy effects clearly affect objective measures and physiological variables in other domains such as pain processing and motor performance [1–2, 5, 17, 25], a possible explanation could be that objective measures in cognitive performance are simply not susceptible to any kind of expectancy manipulation. However, other types of expectancies such as stereotypes and self-efficacy have consistent and well-documented effects on cognitive performance in academic tasks [13–15, 26–27] which renders such a general non-susceptibility unlikely. Nevertheless, a possible, important factor facilitating placebo effects in somatic measures is the participants’ ability to directly attend to the perceived physiological changes and their consequences. Indeed, somatic focus has been reported to influence the effectivity of placebo instruction, i.e., participants who focused more on their feelings and bodily reactions reported more placebo symptoms [28–29]. In cognitive performance, it is more difficult for the participants to perceive possible instruction effects which could reduce the effectiveness of the instructions in cognitive tasks.

Another explanation refers to the type of cognitive task that is investigated. Stereotype threat, for example, seems to affect mostly tasks that rely heavily on working memory [14, 27]. We chose the Flanker task in this study instead because it easily allows the conditioning procedure we sought to implement to maximize possible expectancy effects. However, since the Flanker task is a rather basic interference task targeting cognitive control and flexibility, one could argue that the absence of an expectancy effect is simply due to task choice and that expectancy effects could easily emerge in tasks relying on even higher cognitive functions such as working memory. To pursue this question, we conducted two more experiments using a working memory task that is well-established in research on stereotype threat [27, 30], evoking positive or neutral/negative performance expectancy in participants (S1 File). To further ensure that the experimental manipulation was not responsible for the absence of expectancy effects, we chose two different expectancy inductions for these follow-up experiments: a plausible story on effects of “body posture feedback” as well as a more direct, medical approach during which participants used two nasal sprays either labeled as a cognitive enhancer or as an inactive substance (both nasal sprays contained a saline solution). Despite the change in experimental task and expectancy manipulation, we found no expectancy effects in either experiment (S1 File), further supporting the results of this study.

Which factors could underlie the absence of expectancy effects in objective measures in cognitive performance then? One possibility is that cognitive enhancement is often sought after in situations of high intrinsic motivation, e.g., during exams or for important intellectual challenges [11], and that individuals taking them are already highly convinced of their effect. These aspects are difficult to replicate in a laboratory setting. Indeed, previous research suggests that the expectation of cognitive enhancement can even lead to *worse* results in the laboratory [18], maybe hinting at decreasing motivation in participants to “give their best” in situations of cognitive enhancement. This is unlikely to be a factor in real life situations as intrinsic motivation to perform well is thought to be very high when cognitive enhancers are voluntarily taken of one’s own accord. In this study, we tried to keep motivation high across all experimental phases by including monetary compensation as reward for good performance in all conditions. However, especially the RT data during the conditioning phase indicate that participants did not keep their performance stable, but adapted it primarily to the needs of the task, i.e., they were faster when the task became more difficult (nocebo condition) and slower when the task became easier (placebo condition).

Finally, another possible explanation lies in the expectancy manipulation itself. When expectancies about cognitive performance are manipulated, the process of the expectancy manipulation is usually rather subtle. Sometimes, participants are given actual information about the expectancies in form of a short written statement [31], in other cases the tests were, for example, simply presented as diagnostic of intellectual ability or as known to reveal “gender differences” [15, 32]. In this study, we gave detailed information about the mechanisms and effects of the frequency stimulation and explicitly pointed out which performance effects the participants should expect during which experimental block, similarly to placebo research in the medical domain. However, some researchers argue that conscious awareness of experimental manipulations might attenuate or even reverse effects, for example in social priming [33]. In this case, a more subtle expectancy manipulation could lead to different results in future studies on this matter.

Irrespective of whether or not cognitive placebo and nocebo effects also exist in objective measures, our results clearly show that the subjective perception of cognitive performance is strongly affected by expectancy effects, i.e., individuals believed their performance was improved even if it actually was not. This is another example of a clear dissociation between actual objective measures and simultaneous subjective perception [18–19]. But which process might actually mediate the observed effects on subjective perception? A possible candidate mechanism might be a confirmation bias [34], i.e., a tendency to attend specifically to information that is consistent with one’s expectations. In this context, a confirmation bias could have rendered participants more prone to recall instances of successful performance for the placebo instruction and more instances of unsuccessful performance for the nocebo instruction. A second possibility would be that the participant’s expected ability or anticipated effort to perform the task affected the perception of own performance. Indeed, an influence of anticipated effort has been documented to affect the perception of action-related stimuli (“ability scaling”; [35–36]). Whether or not such an impact might mediate also the observed effects on subjective perception of own performance seems to be a promising direction for future research, as are possible contributions of suggestibility as well as of recall and report biases.

These results emphasize that cognitive improvements that have been discussed as possible placebo effects such as the positive impact of video gaming on cognitive measures (e.g., [37]; for a review of gaming effects on cognitive measures, see [38]) could very well mirror true effects. Moreover, this finding supports the idea that, while expectancy effects can arise for physiological or objective measures in specific domains (such as pain processing) or under specific environmental circumstances (such as stereotype threat), they primarily affect the participants’ or patients’ subjective perception in other domains.

## Supporting Information

S1 File Follow-up Experiments I and II. Description and illustration of the methods and results of Follow-up Experiments I and II, designed to study whether the type of cognitive task or the expectancy manipulation were responsible for the lack of expectancy effects in objective measures in the main experiment.
(PDF)

## References

[1] Colloca L, Benedetti F (2005) Placebos and painkillers: is mind as real as matter? Nat Rev Neurosci 6: 545–552. 15995725 10.1038/nrn1705

[2] Tracey I (2010) Getting the pain you expect: mechanisms of placebo, nocebo and reappraisal effects in humans. Nat Med 16: 1277–1283. 10.1038/nm.2229 20948533

[3] de la Fuente-Fernández R, Ruth TJ, Sossi V, Schulzer M, Calne DB, Stoessl AJ (2001) Expectation and dopamine release: mechanism of the placebo effect in Parkinson's disease. Science 293: 1164–1166. 11498597 10.1126/science.1060937

[4] Mora MS, Nestoriuc Y, Rief W (2011) Lessons learned from placebo groups in antidepressant trials. Philos Trans R Soc Lond B Biol Sci 366: 1879–1888. 10.1098/rstb.2010.0394 21576145 PMC3130402

[5] Enck P, Bingel U, Schedlowski M, Rief W (2013) The placebo response in medicine: minimize, maximize or personalize? Nat Rev Drug Discov 12: 191–204. 10.1038/nrd3923 23449306

[6] Bingel U (2014) Avoiding nocebo effects to optimize treatment outcome. JAMA 312: 693–694. 10.1001/jama.2014.8342 25003609

[7] Geuter S, Büchel C (2013) Facilitation of pain in the human spinal cord by nocebo treatment. J Neurosci 33: 13784–13790. 10.1523/JNEUROSCI.2191-13.2013 23966699 PMC6618657

[8] Chatterjee A (2009) Is it acceptable for people to take methylphenidate to enhance performance? No. BMJ 338: b1956 10.1136/bmj.b1956 19541706

[9] Harris J (2009) Is it acceptable for people to take methylphenidate to enhance performance? Yes. BMJ 338: b1955 10.1136/bmj.b1955 19541705

[10] Hyman SE (2011) Cognitive enhancement: promises and perils. Neuron 69: 595–598. 10.1016/j.neuron.2011.02.012 21338872

[11] Sahakian B, Morein-Zamir S (2007) Professor's little helper. Nature 450: 1157–1159. 18097378 10.1038/4501157a

[12] Sahakian BJ, Morein-Zamir S (2011) Neuroethical issues in cognitive enhancement. J Psychopharmacol 25: 197–204. 10.1177/0269881109106926 20212064

[13] Bandura A (1997) Self-efficacy: The exercise of control. New York, NY: W. H. Freeman.

[14] Schmader T, Johns M, Forbes C (2008) An integrated process model of stereotype threat effects on performance. Psychol Rev 115: 336–356. 10.1037/0033-295X.115.2.336 18426293 PMC2570773

[15] Steele CM, Aronson J (1995) Stereotype threat and the intellectual test performance of African Americans. J Pers Soc Psychol 69: 797–811. 7473032 10.1037//0022-3514.69.5.797

[16] Volkow ND, Wang GJ, Ma Y, Fowler JS, Wong C, Jayne M, et al (2006) Effects of expectation on the brain metabolic responses to methylphenidate and to its placebo in non-drug abusing subjects. Neuroimage 32: 1782–1792. 16757181 10.1016/j.neuroimage.2006.04.192

[17] Stewart-Williams S, Podd J (2004) The placebo effect: dissolving the expectancy versus conditioning debate. Psychol Bull 130: 324–340. 14979775 10.1037/0033-2909.130.2.324

[18] Looby A, Earleywine M (2011) Expectation to receive methylphenidate enhances subjective arousal but not cognitive performance. Exp Clin Psychopharmacol 19: 433–444. 10.1037/a0025252 21875224 PMC3590067

[19] Wechsler ME, Kelley JM, Boyd IO, Dutile S, Marigowda G, Kirsch I, et al (2011) Active albuterol or placebo, sham acupuncture, or no intervention in asthma. N Engl J Med 365: 119–126. 10.1056/NEJMoa1103319 21751905 PMC3154208

[20] Eippert F, Bingel U, Schoell ED, Yacubian J, Klinger R, Lorenz J, et al (2009) Activation of the opioidergic descending pain control system underlies placebo analgesia. Neuron 63: 533–543. 10.1016/j.neuron.2009.07.014 19709634

[21] Amanzio M, Benedetti F (1999) Neuropharmacological dissection of placebo analgesia: expectation-activated opioid systems versus conditioning-activated specific subsystems. J Neurosci 19: 484–494. 9870976 10.1523/JNEUROSCI.19-01-00484.1999 PMC6782391

[22] Colloca L, Benedetti F (2006) How prior experience shapes placebo analgesia. Pain 124: 126–133. 16701952 10.1016/j.pain.2006.04.005

[23] Eriksen BA, Eriksen CW (1974) Effects of noise letters upon the identification of a target letter in a nonsearch task. Percept Psychophys 16(1): 143–149.

[24] Rouder JN, Speckman PL, Sun D, Morey RD, Iverson G (2009) Bayesian *t* tests for accepting and rejecting the null hypothesis. Psychon Bull Rev 16: 225–237. 10.3758/PBR.16.2.225 19293088

[25] Schwarz KA, Sprenger C, Hidalgo P, Pfister R, Diekhof EK, Büchel C (under revision) The tougher sex: How stereotypes affect pain. 10.1038/s41598-019-45044-y PMC6565709 31197222

[26] Pajares F (1996) Self-efficacy beliefs in academic settings. Rev Educ Res 66: 543–578.

[27] Beilock SL, Rydell RJ, McConnell AR (2007) Stereotype threat and working memory: mechanisms, alleviation, and spillover. J Exp Psychol Gen 136: 256–276. 17500650 10.1037/0096-3445.136.2.256

[28] Geers AL, Helfer SG, Weiland PE, Kosbab K (2006) Expectations and placebo response: a laboratory investigation into the role of somatic focus. J Behav Med 29(2): 171–178. 16374671 10.1007/s10865-005-9040-5

[29] Price DD, Finniss DG, Benedetti F (2008) A comprehensive review of the placebo effect: recent advances and current thought. Annu Rev Psychol 59: 565–590. 17550344 10.1146/annurev.psych.59.113006.095941

[30] Krendl AC, Richeson JA, Kelley WM, Heatherton TF (2008) The negative consequences of threat: a functional magnetic resonance imaging investigation of the neural mechanisms underlying women's underperformance in math. Psychol Sci 19: 168–175. 10.1111/j.1467-9280.2008.02063.x 18271865

[31] Wraga M, Duncan L, Jacobs EC, Helt M, Church J (2006) Stereotype susceptibility narrows the gender gap in imagined self-rotation performance. Psychon Bull Rev 13: 813–819. 17328378 10.3758/bf03194002

[32] Spencer SJ, Steele CM, Quinn DM (1999) Stereotype threat and women’s math performance. J Exp Soc Psychol 35: 4–28.

[33] Dijksterhuis A (2014) Welcome back theory! Perspect Psychol Sci 9: 72–75. 26173242 10.1177/1745691613513472

[34] Nickerson RS (1998) Confirmation bias: A ubiquitous phenomenon in many guises. Rev Gen Psychol 2:175–220.

[35] Kirsch W, Königstein E, Kunde W (2014) Hitting ability and perception of object's size: Evidence for a negative relation. Atten Percept Psychophys, 76: 1752–1764. 10.3758/s13414-014-0685-4 24811043

[36] Proffitt DR, Linkenauger SA (2013) Perception viewed as a phenotypic expression In Prinz W. (Ed.), Tutorials in Action Science (pp. 171–198). Cambridge: MIT Press.

[37] Boot WR, Simons DJ, Stothart C, Stutts C (2013) The pervasive problem with placebos in psychology. Perspect Psychol Sci 8: 445–454. 26173122 10.1177/1745691613491271

[38] Green CS, Bavelier D (2012) Learning, attentional control, and action video games. Curr Biol 22: R197–R206. 10.1016/j.cub.2012.02.012 22440805 PMC3461277

[39] Pfister R, Janczyk M (2013) Confidence intervals for two sample means: Calculation, interpretation, and a few simple rules. Adv Cogn Psychol 9: 74–80. 10.2478/v10053-008-0133-x 23826038 PMC3699740

[40] Loftus GR, Masson ME (1994) Using confidence intervals in within-subject designs. Psychon Bull Rev 1: 476–490. 10.3758/BF03210951 24203555

