# Supplementary material for: Cognition and the Placebo Effect – Dissociating Subjective Perception and Actual Performance
Source: PLoS One. 2015 Jul 6;10(7):e0130492. doi: 10.1371/journal.pone.0130492 (PMC4493024; doi:10.1371/journal.pone.0130492)
Supplement: S1 File — Description and illustration of the methods and results of Follow-up Experiments I and II, designed to study whether the type of cognitive task or the expectancy manipulation were responsible for the lack of expectancy effects in objective measures in the main experiment. (PDF) [file pone.0130492.s001.pdf]

Cognition and the Placebo Effect

# – Supporting Information –

General Instruction

Two possible explanations for the absence of an expectancy effect in objective measures in our main experiment concern (1) the type of task that was used, i.e., maybe such effects appear only in higher cognitive functions such as tasks based on working memory, instead of simple reaction time tasks, and (2) the expectancy manipulation itself. To pursue these questions, we conducted two follow-up experiments that employed specific arithmetic tasks which heavily rely on working memory capacity and were already successfully introduced in research on stereotype threat [26-27]. Moreover, we used two different expectancy manipulations: in the first experiment, we told participants that specific body postures would enhance or impair cognitive performance, respectively, via a body feedback process. In the second experiment, we used a more direct approach by pretending to administer a cognitive enhancer or a saline solution to the participants, when in reality both probes contained saline solution.

Follow-up Experiment I

Materials and Methods

Participants

We recruited 41 individuals (24 female; mean age 26.39 years ± 0.83 $SE_{M;}$ mean age [female participants] 26.63 years ± 1.19 SE_M; mean age [male participants] 26.06 years ± 1.14 SE_M). All participants received payment as compensation. Exclusion criteria involved neurological or neuropsychiatric diseases, current medication, or substance abuse. The study was approved by the Ethics Committee of the Medical Council of Hamburg and all participants gave written consent in accordance with the Declaration of Helsinki.

Expectancy Manipulation

Participants were informed at the beginning of the experiment that they would take part in a study investigating body posture feedback on cognitive performance. Half of the participants were instructed that a tense body posture would increase cognitive performance (placebo condition), whereas a relaxed body posture would decrease cognitive performance (nocebo condition) via body feedback mechanisms. The other half of the participants were instructed that

a relaxed body posture would lead to better cognitive performance (placebo condition), whereas a tense body posture would impair cognitive performance (nocebo condition). Instructions were randomized across participants.

Experimental Procedure

As dependent variables, we measured reaction times (RTs) and success rates (SRs) in a modular arithmetic task adapted from the literature on stereotype threat [26]. This task was shown to be sensitive to stereotype-relevant instructions and relies heavily on working memory capacity [26]. Participants saw equations on the screen and had to decide whether or not the equation was correct. Equations were created in three difficulty levels (easy, medium, hard). To assure a high motivation throughout the experiment, we instructed the participants at the very beginning that the amount of money they would receive for study compensation would be increased proportionally to their performance across all experimental blocks.

All participants first completed a short introductory block of 12 trials (4 easy, 4 medium, 4 hard; 6 correct, 6 incorrect) to become acquainted with the task. Control of the experimental timing and the stimulus presentation throughout the experiment was achieved using Presentation 16.4, NeuroBehavioral Systems (Albany, CA, USA). Each trial started with a fixation cross presented on a computer screen for 500ms, followed by an equation. The equation was presented until participants responded with a button press. Half of the participants were instructed to respond with the left arrow key on the computer keyboard when the equation was correct and with the right arrow key if the equation was incorrect, whereas the other half was instructed with the opposite mapping. A feedback screen was shown for 1000ms informing them if their answer had been correct or not. The next trial started after an inter-trial interval of 1000ms.

After the introductory block, participants completed two test blocks, one test block in the placebo, one in the nocebo condition. Condition order was randomized across participants. Each test block consisted of 54 trials, 18 easy, 18 medium, and 18 hard; 27 equations were correct, 27 were incorrect. No equations were repeated within a participant.

At the end of the experiment, participants were debriefed about the actual study purpose and were asked to indicate on a 7-point scale whether they had believed the previous instruction. Three participants (1 female) had to be excluded, because they did not believe our expectancy manipulation.

Behavioral data analyses

Behavioral data were analyzed using SPSS 20 (IBM, Armonk, NY, USA). We calculated SRs for each participant and the mean RT of all successfully completed trials for each participant, separately for the placebo and nocebo conditions. The introductory block was not included in the calculations. We then performed an ANOVA with the within-subjects factors

expectancy (placebo vs. nocebo) and difficulty (easy vs. medium vs. hard) for the RT and SR data and performed paired *t*-tests as follow-up analyses.

Results

The expectancy instruction did not have any significant effects on performance in the modular arithmetic task on any measure (SR: $F(1,37)=2.43$, $p=.128$; RT: $F(1,37)<1$; Fig. A). In contrast, the difficulty level had strong effects on both measures, i.e., the higher the difficulty level, the lower the SR and the slower the RT, irrespective of the expectancy condition (SR: $F(2,74)=44.51$, $p<.001$, $\eta_p^2=0.55$; RT: $F(2,74)=150.71$, $p<.001$, $\eta_p^2=0.80$, $\varepsilon=.614$, Greenhouse-Geisser corrected for violations of sphericity; Fig. A).

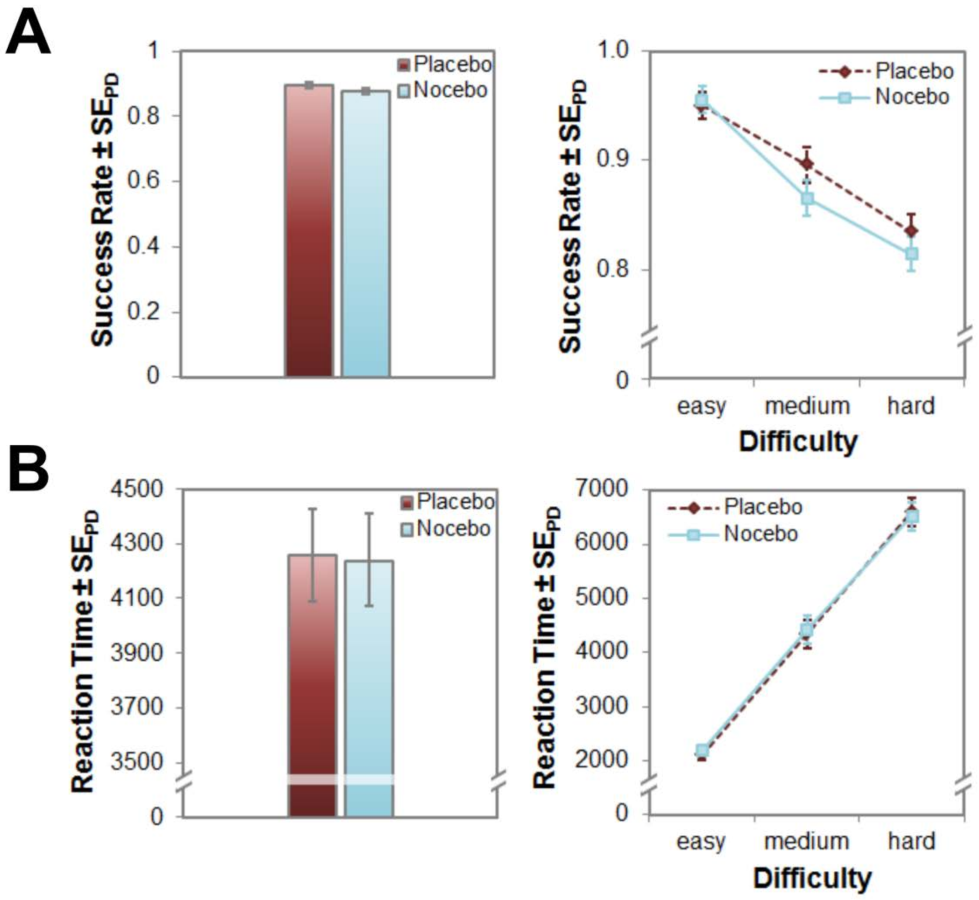

Fig. A. (A) SRs and **(B)** RTs for placebo and nocebo conditions in the modular arithmetics task of Follow-up Experiment I. No effects of expectancy emerged. Error bars indicate standard errors of paired differences [33]. The expectancy instruction did not have any significant effects on SRs or RTs, whereas the difficulty level had strong effects on both measures. No interaction of expectancy and difficulty emerged in either SRs or RTs.

All follow-up paired *t*-tests comparing SRs and RTs in the different difficulty levels were significant (all *p*s<.001). However, no interaction of expectancy and difficulty emerged in either SRs or RTs (SR: $F(2,74)=2.00$, $p=.143$; RT: $F<1$), indicating that the expectancy manipulation simply had no statistically valid effect on any objective measure (Fig. A).

Follow-up Experiment II

Materials and Methods

Participants

We recruited 37 individuals (17 female; mean age 25.44 years ± 0.85 $SE_{M;}$ mean age [female participants] 24.64 years ± 0.63 SE_M; mean age [male participants] 26.10 years ± 1.42 SE_M). All participants received payment as compensation. Exclusion criteria involved neurological or neuropsychiatric diseases, current medication, or substance abuse. The study was approved by the Ethics Committee of the Medical Council of Hamburg and all participants gave written consent in accordance with the Declaration of Helsinki.

Expectancy Manipulation

Participants were informed at the beginning of the experiment that they would take part in a study in which a cognitive enhancer, oxytocin, was used and applied via nasal sprays. We also informed them about diverse positive effects of oxytocin on cognitive performance. They were told that they would take part in three blocks, at first one short block without any medication, then one block with oxytocin (placebo condition) and one block with an inactive substance (control condition). The order of the expectancy conditions were randomized across participants. The nasal sprays were labeled accordingly, although all nasal sprays contained a saline solution, irrespective of labeling. When using the nasal sprays, all participants were instructed to spray four times, twice in each nostril, and to wait for 10 minutes after application before starting the experimental procedure to allow the "medication to become effective". At least 40 minutes lay between each nasal spray application to allow the "medication to lose effectiveness" before applying the next nasal spray.

Experimental Procedure

As dependent variables, we measured reaction times (RTs) and success rates (SRs) in an arithmetic task including modular arithmetic and basic arithmetic tasks adapted from the literature on stereotype threat [26-27]. This task was shown to be sensitive to stereotype-relevant instructions and relies heavily on working memory capacity [26-27]. Participants saw equations on the screen and had to decide whether or not the equation was correct. Equations

were created in three difficulty levels (easy, medium, hard). To assure a high motivation throughout the experiment, we instructed the participants at the very beginning that the amount of money they would receive for study compensation would be increased proportionally to their performance across all experimental blocks.

All participants first completed one experimental phase without any medication application. It started with a short introductory block of 12 trials (4 easy, 4 medium, 4 hard; 6 correct, 6 incorrect), followed by a short training block of 16 trials (4 easy, 8 medium, 4 hard; 8 correct, 8 incorrect), and the actual test phase of 24 trials (8 easy, 8 medium, 8 hard; 12 correct, 12 incorrect). This was intended to get participants acquainted with the task and the block structure. After the training and after the test block, participants were given feedback about their performance.

Control of the experimental timing and the stimulus presentation throughout the experiment was achieved using Presentation 16.4, NeuroBehavioral Systems (Albany, CA, USA). Each trial started with a fixation cross presented on a computer screen for 500ms, followed by an equation. The equation was presented until participants responded with a button press, up to a maximum of 7000ms. Half of the participants were instructed to respond with the left arrow key on the computer keyboard when the equation was correct and with the right arrow key if the equation was incorrect, whereas the other half was instructed with the opposite mapping. The next trial started after an inter-trial interval of 1000ms.

After the first experimental phase, participants either first completed the oxytocin phase (placebo condition) and then the "inactive substance" phase (control condition) or vice versa; condition order was randomized across participants. The oxytocin phase started with a short introductory block of 6 trials (2 easy, 2 medium, 2 hard; 3 correct, 3 incorrect), followed by a training block of 16 trials (8 easy, 4 medium, 4 hard; 8 correct, 8 incorrect). To increase credibility of our previous instruction, we increased the amount of easy equations in this training block, and showed a higher overall performance during feedback after the training block by adding 12.5% to the participants' actual success rate (up to a maximum of 94%). The subsequent test block consisted of 48 trials equally distributed across all difficulty levels (16 easy, 16 medium, 16 hard; 24 correct, 24 incorrect); feedback at the end of the test block, however, was again manipulated to improve the participants' performance by 12.5% (up to a maximum of 96%). The "inactive substance" phase also started with a short introductory block of 6 trials (2 easy, 2 medium, 2 hard; 3 correct, 3 incorrect), followed by a training block of 16 trials (4 easy, 8 medium, 4 hard; 8 correct, 8 incorrect), and by a test block of 48 trials (16 easy, 16 medium, 16 hard; 24 correct, 24 incorrect). Feedback was again given at the end of the training and at the end of the test block, without any experimental manipulation. No equation in the training or test blocks was repeated within a participant.

At the end of the experiment, participants were debriefed about the actual study purpose and were asked to indicate on a 7-point scale whether they had believed the previous instruction. Nine participants (2 female) had to be excluded, because they did not believe our expectancy manipulation.

Behavioral data analyses

Behavioral data were analyzed using SPSS 20 (IBM, Armonk, NY, USA). We calculated SRs for each participant and the mean RT of all successfully completed trials for each participant, separately for the placebo and nocebo conditions. We only included the test blocks of the oxytocin and the "ineffective substance" blocks in our calculations. We then performed an ANOVA with the within-subjects factors expectancy (placebo vs. nocebo) and difficulty (easy vs. medium vs. hard) for the RT and SR data and performed paired *t*-tests as follow-up analyses.

Results

The results of this experiment precisely mirror the results of Follow-up Experiment I. Again, no expectancy effect emerged in either objective measure (SR: $F<1$; RT: $F<1$; Fig. B), but difficulty levels strongly affected SRs and RTs, as expected (SR: $F(2,54)=68.71$, $p<.001$, $\eta_p^2=0.72$; RT: $F(2,54)=171.99$, $p<.001$, $\eta_p^2=0.86$, $\varepsilon=.709$, Greenhouse-Geisser corrected for violations of sphericity; Fig. B).

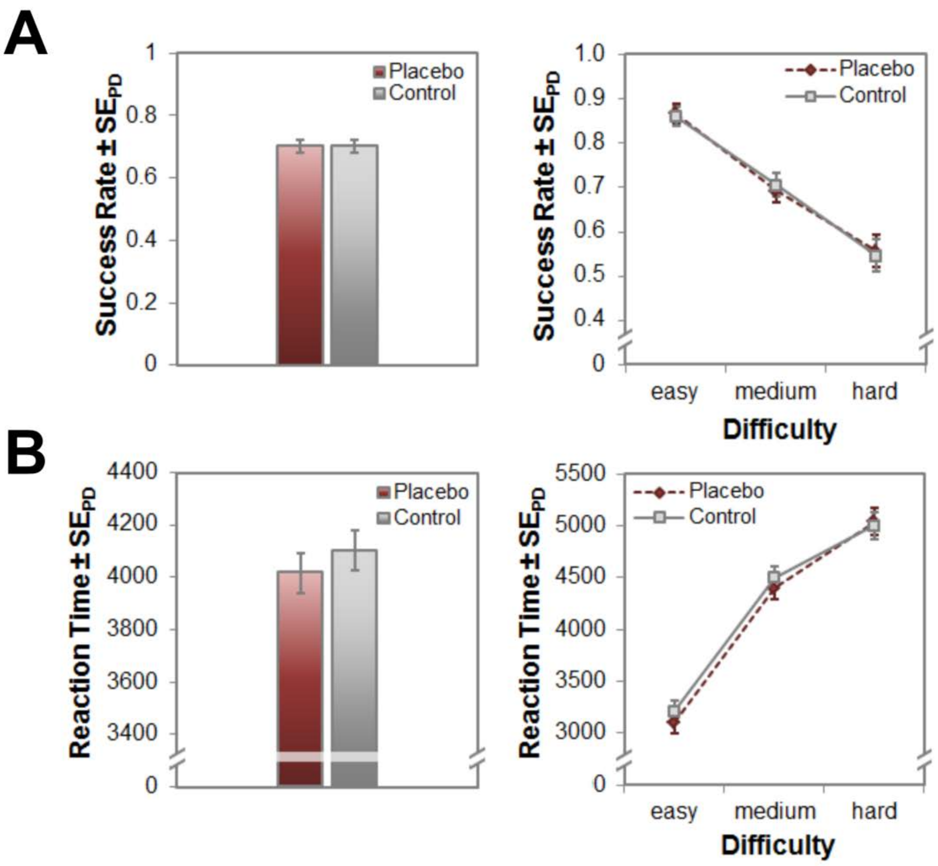

Fig. B. (A) SRs and **(B)** RTs for placebo and control conditions in the arithmetic task of Follow-up Experiment II. No effects of expectancy emerged. Error bars indicate standard errors of the paired differences [33]. The expectancy instruction did not have any significant effects on SRs

or RTs, whereas the difficulty level had strong effects on both measures. No interaction of expectancy and difficulty emerged in either SRs or RTs.

SRs and RTs were significantly different between all difficulty levels, as follow-up paired *t*-tests indicated (all $ps<.001$). The expectancy x difficulty interaction did not approach significance for either measure (SR: $F<1$; RT: $F<1$). These results again indicate that the expectancy manipulation had no effect on any objective measure (Fig. B).

Conclusion

Our follow-up experiments were designed to investigate whether expectancy effects in objective measures in placebo or nocebo conditions did not emerge in our main experiment, because of our task choice or because of the applied expectancy manipulation. Therefore, we employed an arithmetic task that heavily relies on working memory capacity and that was already shown to be sensitive to instructions in research on stereotype threat. We also chose two different forms of expectancy manipulations to ensure that this factor was not crucial for our results. Both follow-up experiments showed very similar patterns: whereas difficulty levels had strong effects on success rates and reaction times, expectancy manipulations did not affect cognitive performance in any objective measure. This indicates that the results of our main experiment are valid in that objective measures targeting cognitive performance seem to remain unaffected by placebo or nocebo instructions; subjective measures, in contrast, were easily manipulated in our main experiment.

An additional observation concerns the reception of placebo instructions in the three different experiments: placebo instructions focusing on “frequency effects” as in the main experiment or on a plausible story without any sham intervention (Follow-up Experiment I) seemed to be more believable to the participants than the cover story allegedly involving an actual drug (Follow-up Experiment II). This seems counterintuitive given that the predominant view is that the more invasive the procedure the more effective the placebo instruction (e.g., [SR1]). However, during Follow-up Experiment II, some participants seemed more skeptical about the precise instructions (especially about when they were given which medication) and suspected deception. This skepticism seemed to be focused on the labelling of medication as “inactive” or “effective” which would explain why it primarily occurred in Follow-up Experiment II. Interestingly, this problem seems to be far more pronounced in the context of cognitive performance, whereas it is negligible in the context of placebo analgesia in our experience.

Supplementary References

SR1. Diener HC (2010). Placebo effects in treating migraine and other headaches. Curr Opin Investig Drugs 11(7): 735-739.

Data

Tab. S1. Reaction times (RTs) for each participant and condition in the test phase of the main experiment.

	Placebo		Nocebo		Control	
Subject	Compatible	Incompatible	Compatible	Incompatible	Compatible	Incompatible
1	359	391	352	394	361	388
2	387	454	363	453	371	458
3	391	478	398	475	377	470
4	365	435	362	427	357	431
5	359	475	379	460	378	427
6	352	379	317	346	326	323
7	372	426	365	440	367	433
8	324	381	315	356	310	374
9	387	467	386	522	374	460
10	399	476	411	499	406	466
11	390	450	372	446	374	466
12	351	395	355	383	362	419
13	338	395	353	403	344	400
14	337	380	320	361	319	360
15	379	469	364	462	368	489
16	363	452	375	461	357	447
17	355	459	363	460	373	430
18	340	387	337	399	340	393
19	386	436	368	421	381	440
20	344	438	346	426	339	451
21	381	435	375	442	376	436
22	368	458	373	448	374	462
23	318	379	307	337	319	359
24	358	447	365	449	353	454
25	406	506	399	498	418	508
26	399	441	392	467	393	460
27	333	348	317	332	330	337
28	433	532	424	537	428	524
29	350	424	341	409	354	428
30	361	445	368	437	364	425
31	344	376	342	384	346	380
32	363	429	361	414	367	454
33	363	433	360	422	355	418
34	351	401	350	409	351	424
35	377	396	333	391	347	409
36	323	387	327	376	333	382

Tab. S2. Success rates (SRs) for each participant and condition in the test phase of the main experiment.

	Placebo		Nocebo		Control	
Subject	Compatible	Incompatible	Compatible	Incompatible	Compatible	Incompatible
1	0.66	0.26	0.68	0.19	0.67	0.26
2	0.94	0.30	1.00	0.13	0.94	0.18
3	0.97	0.25	0.90	0.16	1.00	0.30
4	0.84	0.13	0.89	0.13	0.90	0.10
5	1.00	0.33	0.97	0.35	0.97	0.43
6	0.68	0.12	0.62	0.18	0.59	0.39
7	1.00	0.54	1.00	0.41	0.97	0.37
8	0.94	0.25	0.97	0.34	0.92	0.21
9	0.78	0.05	0.71	0.00	0.80	0.11
10	0.92	0.15	0.92	0.06	0.80	0.17
11	0.88	0.50	0.97	0.50	1.00	0.46
12	0.92	0.21	0.80	0.33	0.82	0.13
13	0.92	0.44	0.83	0.30	0.94	0.29
14	0.89	0.29	0.97	0.45	0.89	0.36
15	0.91	0.11	1.00	0.17	0.98	0.15
16	1.00	0.31	0.97	0.28	1.00	0.27
17	0.97	0.17	0.85	0.06	0.81	0.33
18	0.91	0.28	0.96	0.27	0.95	0.30
19	0.94	0.29	0.94	0.53	0.97	0.37
20	1.00	0.06	1.00	0.15	0.97	0.06
21	0.95	0.25	0.92	0.13	0.79	0.24
22	0.98	0.17	1.00	0.15	0.97	0.06
23	0.97	0.31	1.00	0.32	0.95	0.36
24	0.91	0.22	0.97	0.24	0.93	0.24
25	0.90	0.21	0.90	0.18	0.77	0.13
26	0.74	0.16	0.83	0.10	0.72	0.12
27	0.73	0.24	0.79	0.31	0.78	0.27
28	0.92	0.26	1.00	0.31	0.91	0.31
29	1.00	0.66	0.97	0.61	1.00	0.42
30	0.95	0.03	0.84	0.15	0.95	0.30
31	0.81	0.45	0.89	0.43	0.82	0.50
32	1.00	0.11	0.97	0.25	1.00	0.17
33	1.00	0.37	1.00	0.47	1.00	0.33
34	0.83	0.18	0.86	0.19	0.76	0.10
35	0.74	0.28	0.88	0.30	0.94	0.23
36	0.97	0.16	0.94	0.06	0.94	0.09

Tab. S3. Ratings for each participant and condition of the main experiment.

Subject	Placebo	Nocebo	Control
1	8	3	4
2	6	6	6
3	7	5	6
4	6	7	3
5	6	4	6
6	8	1	6
7	5	5	3
8	6	3	4
9	6	3	6
10	1	1	8
11	8	3	6
12	7	2	3
13	4	3	6
14	8	4	1
15	6	5	3
16	3	3	6
17	7	4	6
18	6	4	5
19	7	7	3
20	4	2	6
21	5	1	7
22	3	4	3
23	7	5	5
24	6	7	4
25	5	7	2
26	5	4	3
27	5	2	5
28	6	5	2
29	8	3	3
30	5	5	5
31	5	2	1
32	7	4	5
33	8	4	7
34	8	3	4
35	6	3	5
36	7	5	5

Tab. S4. Reaction times (RTs) for each participant and condition in Follow-up Experiment I.

	Placebo			Nocebo		
Subject	easy	medium	hard	easy	medium	hard
1	1866	6887	9053	2750	4208	12369
2	1798	3422	6640	1705	2717	6081
3	2439	4201	6581	1686	3743	4799
4	1716	3465	6000	1822	3654	6231
5	1571	4468	6499	2064	6449	8011
6	1880	3607	5595	1760	4081	4924
7	2019	4549	4549	1908	3244	4430
8	1581	2611	3483	1613	2854	2850
9	2275	3746	6041	2463	4116	5087
10	1618	2426	5430	2134	3450	6407
11	1496	2320	4125	1728	2927	4077
12	2597	4386	5456	1916	3372	6894
13	1349	3430	4009	1676	2979	4258
14	1314	2259	3321	1252	2065	3832
15	2033	5964	7722	1641	4026	6266
16	1412	2961	4467	1361	2354	3834
17	2953	10184	13929	3769	8873	10606
18	1760	2762	4047	1537	2974	4435
19	3016	4431	6202	5363	10037	9311
20	2060	4584	6630	2010	3378	5445
21	2186	3082	5218	2056	4565	5286
22	2535	4430	5829	2129	4418	6135
23	1765	3829	6383	2057	5286	6546
24	1476	3323	6440	1140	2522	3615
25	2835	5711	11955	4460	8655	11659
26	1974	5126	10094	2291	6348	9293
27	2352	7510	10766	2231	5292	10115
28	1615	3610	5114	1531	3098	4252
29	2781	4768	7783	2613	5298	9218
30	3443	9817	14714	2572	6647	11208
31	1701	2869	6531	1797	2896	4613
32	2300	4471	4339	2464	4669	7071
33	1838	2333	3670	1885	3928	4190
34	1963	4102	8967	1936	4380	6568
35	1697	3049	4098	1329	2194	3972
36	3772	6549	7528	3839	8713	10399
37	3641	4613	5453	2779	4240	6396
38	1499	3390	5940	2098	3585	6874

Tab. S5. Success rates (SRs) for each participant and condition in Follow-up Experiment I.

	Placebo			Nocebo		
Subject	easy	medium	hard	easy	medium	hard
1	0.94	0.89	0.83	0.94	0.89	0.72
2	1.00	0.83	1.00	0.94	0.94	0.83
3	1.00	0.83	0.72	0.89	0.83	0.78
4	0.94	0.94	0.89	0.94	0.94	1.00
5	0.94	1.00	1.00	0.94	0.78	0.94
6	1.00	1.00	0.83	1.00	1.00	0.89
7	0.89	0.94	0.83	0.94	0.89	0.89
8	1.00	0.67	0.61	0.83	0.78	0.61
9	0.89	0.83	0.67	0.94	0.83	0.78
10	0.94	0.83	0.78	1.00	0.89	0.78
11	1.00	1.00	0.89	0.94	0.89	0.89
12	0.89	0.94	0.78	1.00	0.94	0.89
13	0.94	0.94	0.89	0.94	0.94	0.89
14	0.94	1.00	0.94	0.94	1.00	0.89
15	0.94	0.94	0.94	1.00	1.00	0.89
16	0.94	0.94	0.89	1.00	0.83	0.89
17	1.00	1.00	0.89	1.00	0.89	0.78
18	1.00	0.94	0.78	0.94	1.00	0.83
19	1.00	0.94	0.61	0.83	0.56	0.44
20	1.00	0.89	0.83	1.00	0.89	0.89
21	0.94	0.78	0.89	1.00	0.72	0.83
22	0.94	0.78	0.72	0.94	0.67	0.50
23	0.94	0.94	0.83	1.00	0.94	0.78
24	1.00	1.00	0.89	0.89	0.89	0.78
25	0.94	0.72	0.67	0.94	0.72	0.94
26	1.00	1.00	1.00	1.00	1.00	0.94
27	1.00	0.89	0.94	1.00	0.89	0.78
28	0.94	0.94	0.83	1.00	1.00	0.78
29	1.00	1.00	0.94	1.00	1.00	0.94
30	0.89	0.83	0.89	0.94	0.94	0.78
31	0.94	0.94	0.89	1.00	0.89	0.83
32	0.83	0.83	0.78	1.00	0.67	0.67
33	1.00	0.94	0.89	0.94	1.00	1.00
34	0.78	1.00	0.89	0.94	0.83	0.94
35	1.00	0.89	0.89	0.94	0.78	0.83
36	0.94	0.67	0.67	0.89	0.61	0.56
37	0.83	0.72	0.78	0.94	0.83	0.78
38	1.00	0.89	0.72	1.00	0.78	0.78

Tab. S6. Reaction times (RTs) for each participant and condition in Follow-up Experiment II.

	Placebo			Control		
Subject	easy	medium	hard	easy	medium	hard
1	2713	3462	4311	2821	4338	4156
2	1999	3500	4840	2574	4373	5403
3	2762	4053	5515	3757	4663	5227
4	2791	3872	4422	2694	4362	4960
5	2940	4463	5251	3611	5014	4999
6	2374	3428	4105	2539	3893	3886
7	2921	4457	4227	2947	4427	4915
8	3026	3607	4623	2391	3838	4876
9	2968	4824	5512	2772	4396	5381
10	3496	4785	5265	3364	4675	5075
11	4439	4202	3949	4681	4573	4525
12	3002	4751	5309	2784	3290	5304
13	2928	4135	4967	3365	5197	5136
14	3458	4587	6314	3083	4069	5142
15	2678	5112	5612	3421	5384	5755
16	3076	3822	4283	2792	3939	4621
17	3299	4698	4826	4426	5111	4519
18	3461	4093	5104	3233	4406	4549
19	2428	4747	5436	2870	4466	4906
20	4041	5373	4512	3892	4992	5372
21	3307	4935	4661	3043	4259	5867
22	4030	5605	6243	3475	4582	3895
23	3082	4822	5694	3368	4736	5240
24	2779	4515	5350	3348	5174	5598
25	4457	4687	5575	3290	4615	4636
26	3043	4911	5124	3762	4867	5356
27	2605	3694	5333	2371	4544	5775
28	2763	4056	4716	3303	3893	5073

Tab. S7. Success rates (SRs) for each participant and condition in Follow-up Experiment II.

	Placebo			Control		
Subject	easy	medium	hard	easy	medium	hard
1	0.75	0.81	0.56	0.75	0.94	0.63
2	1.00	1.00	0.75	1.00	0.94	0.56
3	0.88	0.63	0.50	0.69	0.44	0.38
4	0.94	0.88	0.56	0.69	0.94	0.56
5	0.88	0.63	0.44	1.00	0.75	0.44
6	1.00	0.88	0.81	1.00	0.81	0.69
7	1.00	0.88	0.50	0.88	0.63	0.69
8	1.00	0.88	0.69	0.94	0.69	0.38
9	0.94	0.63	0.56	0.94	0.63	0.50
10	0.69	0.50	0.50	0.88	0.63	0.56
11	0.63	0.56	0.38	0.56	0.63	0.63
12	1.00	0.88	0.69	1.00	0.81	0.88
13	0.69	0.69	0.50	0.88	0.56	0.56
14	0.88	0.75	0.13	1.00	0.88	0.63
15	0.88	0.50	0.56	0.88	0.44	0.38
16	1.00	0.81	0.63	1.00	0.94	0.75
17	0.88	0.75	0.75	0.56	0.75	0.50
18	0.69	0.56	0.69	0.69	0.44	0.63
19	0.94	0.94	0.75	1.00	0.81	0.63
20	0.81	0.50	0.38	0.81	0.56	0.44
21	0.94	0.75	0.81	0.94	0.69	0.69
22	0.81	0.38	0.25	1.00	0.56	0.19
23	0.75	0.44	0.44	0.69	0.63	0.56
24	0.75	0.69	0.81	0.69	0.69	0.44
25	0.81	0.44	0.56	0.81	0.69	0.31
26	0.88	0.44	0.50	0.81	0.69	0.63
27	0.94	0.81	0.50	1.00	0.94	0.50
28	0.88	0.75	0.38	1.00	0.63	0.56
